# Shoulder Replacement in the Elderly with Anatomic versus Reverse Total Prosthesis? A Prospective 2-Year Follow-Up Study

**DOI:** 10.3390/jcm11030540

**Published:** 2022-01-21

**Authors:** Maciej J. K. Simon, Jennifer A. Coghlan, Simon N. Bell

**Affiliations:** 1 Melbourne Shoulder and Elbow Centre, 1/80 Beach Road, Sandringham, Melbourne, VIC 3191, Australia; maciej.simon@gmail.com (M.J.K.S.); coghlan@bigpond.net.au (J.A.C.); 2 Department of Orthopaedics and Trauma Surgery, Campus Kiel, University Medical Center Schleswig-Holstein, Arnold-Heller-Str. 3, 24105 Kiel, Germany; 3 Department of Surgery, School of Clinical Sciences, Monash Health, Monash University, Clayton, Melbourne, VIC 3168, Australia

**Keywords:** anatomic total shoulder replacement, reverse total shoulder replacement, Lazarus score, Sirveaux score, older patients, clinical scores

## Abstract

Background: In older patients requiring a total shoulder replacement (TSR) and with an intact rotator cuff, there is currently uncertainty on whether an anatomic TSR (aTSR) or a reverse TSR (rTSR) is best for the patient. This comparison study of same-aged patients aims to assess clinical and radiological outcomes of older patients (≥75 years) who received either an aTSR or an rTSR. Methods: Consecutive patients with a minimum age of 75 years who received an aTSR (*n* = 44) or rTSR (*n* = 51) were prospectively studied. Pre- and post-operative clinical evaluations included the ASES score, Constant score, SPADI score, DASH score, range of motion (ROM) and pain and patient satisfaction for a follow-up of 2 years. Radiological assessment identified glenoid and humeral component osteolysis, including notching with an rTSR. Results: We found postoperative improvement for ROM and all clinical assessment scores for both groups. There were significantly better patient reported outcome scores (PROMs) in the aTSR group compared with the rTSR patients (*p* < 0.001). Both groups had only minor osteolysis on radiographs. No revisions were required in either group. The main complications were scapular stress fractures for the rTSR (*n* = 11) patients and acromioclavicular joint pain for both groups (aTSR = 2; rTSR = 6). Conclusions: This study of older patients (≥75 years) demonstrated that an aTSR for a patient with good rotator cuff muscles can lead to a better clinical outcome and less early complications than an rTSR. Level of evidence: Level II—prospective cohort study.

## 1. Introduction

In shoulder replacement, the type of shoulder prosthesis chosen for a particular patient is based on the underlying pathology, in particular the status of the rotator cuff, the degree of bony erosion, and the biological age of the patient.

Rotator cuff tendons degenerate progressively with increasing age and with secondary wasting of the muscle belly [1,2], and rotator cuff tears are present in almost 50% of the population in their 8th or 9th decade of life [3]. In the other 50% of older patients with an arthritic glenohumeral joint but an intact rotator cuff requiring a total shoulder replacement (TSR), there is at present uncertainty as to which patients would do better with an aTSR. There is a currently an increasing tendency to recommend a reverse total shoulder replacement (rTSR) in patients older than 70 years with an intact rotator cuff [4], which seems to assume that the result of an aTSR vs. an rTSR in this age group is similar.

This study aims to compare the results both clinically and radiologically, and the rate of complications, between a group of patients 75 years or over who had either an aTSR or an rTSR. The hypothesis is that patients with an aTSR have better clinical outcomes than same age patients receiving an rTSR.

## 2. Materials and Methods

### 2.1. Study Protocol and Patients’ Eligibility

The nested study interrogated two existing databases from the same department. These included consecutive patients 75 years or older with either advanced primary glenohumeral osteoarthritis (OA) with or without an intact rotator cuff (RC), or an irreparable rotator cuff tear with minor osteoarthritis, who had a TSR.

Decision on prosthesis type (aTSR or rTSR) depended on the rotator cuff’s status. Initial assessment was made clinically and radiologically with an X-ray and CT scan. If the integrity of the rotator cuff was uncertain, these cases were further assessed with an MRI scan to assess the rotator cuff status, including, if present, tear type (full or partial thickness), extent of muscle atrophy, and degeneration [5,6]. If the patient had osteoarthritis but the RC was intact and not degenerated, with no major muscle atrophy, the decision was made for an aTSR. If the patient had osteoarthritis and the RC was torn or degenerated, including severe fatty atrophy, the decision was made for an rTSR. If imaging demonstrated a massive irreparable RC tear with minor arthritis, an rTSR was indicated.

Inclusion criteria for the group receiving an aTSR were glenohumeral OA and an intact rotator cuff. The criteria for inclusion for the group receiving an rTSR were glenohumeral arthritis with an inadequate rotator cuff for an aTSR (*n* = 25), or a massive irreparable rotator cuff tear (*n* = 26).

Exclusion criteria were age under 75 years before the operation, post fracture/traumatic osteoarthritis, abnormal neurology or inability to comply with the study requirements. Exclusion criterion for performing an aTSR was a full-thickness rotator cuff tear, or inadequate rotator cuff function based on clinical examination and MRI findings. Therefore, all patients with glenohumeral arthritis who had a good rotator cuff had an aTSR, despite their age.

Eligible consecutive patients gave written consent and were prospectively enrolled in the study over the same time period. An identical prospective study protocol was set up for both groups. All patients had a minimum age of 75 years at the time of operation and a minimum follow-up time of 2 years. Written consent for study participation and publication was obtained from all patients. All studies were carried out in accordance with the World Medical Association most recent Declaration of Helsinki.

### 2.2. Clinical Assessments

Preoperatively, patient-reported outcome measures (PROMs), including the American shoulder and elbow surgeons (ASES) score [7,8], shoulder pain and disability index (SPADI) [9,10], disability of the arm, shoulder and hand (DASH) scores [9,11], pain (VAS 0 = no pain-10 = severe pain), were recorded, together with the Constant score (CS) [12,13], and clinical range of motion (ROM) for all patients in the study.

Postoperatively, patients were clinically reviewed at 2-week, 6-week, 3-month, 6-month, 1-year, and 2-year time points. Pain levels and ROM were documented at 3 months, 1 year and 2 years. Active and passive ROM, CS, and PROMs were recorded at 1-year and 2-year follow-up. Satisfaction (scored from 0%—dissatisfied to 100%—totally satisfied) was also recorded.

### 2.3. Radiological Assessments

Radiological assessments were carried out preoperatively (Walch and Favard classification), and postoperatively at day 1, 12 weeks and yearly [14,15,16]. In order to assess both the humeral and glenoid components, radiographs were taken according to a standardized protocol in multiple planes (axillary, true lateral, standard anteroposterior (AP) and true AP view of glenoid with the arm in 20° external and internal rotation). Radiographs were assessed independently and separately by two orthopaedic surgeons (MJKS and MC) not involved in the patient surgeries. Disagreements were referred to a third independent experienced surgeon (HC) for final decision.

The aTSR was radiologically evaluated and assessed in a standard technique for perihumeral component osteolysis with assessment of five zones, as previously described [17]. The glenoid component was assessed using the Lazarus classification quantifying radiolucent lines (RLL) between the cement surrounding the glenoid pegs and bone interface [18].

The rTSR was radiologically assessed in a standard technique for scapular notching according to the classification by Sirveaux et al. for the glenoid component [19,20]. The humeral component was assessed for RLL around the implant in seven different zones as previously described by Levigne et al. and Bell et al. [16,21,22].

### 2.4. Implants and Postoperative Rehabilitation

All shoulders were replaced via a standard deltopectoral approach. For the aTSR, a stemless Affinis^®^ short humeral ceramic head component was utilized in all cases. This was articulated with a double-pegged, cemented, all-polyethylene glenoid component made of standard, not cross-linked, polyethylene (Mathys AG, Bettlach, Switzerland) in 34 cases and more recently in 11 cases with a highly cross-linked polyethylene (HXLPE)—Affinis^®^ Glenoid Vitamys prosthesis (Mathys AG, Bettlach, Switzerland).

For rTSR, a Grammont-style humeral prosthesis was used—Aequalis Reversed II Shoulder System (Tornier SAS (Montbonnot Saint Martin, France) part of Stryker). For the glenoid, a 25 mm baseplate was utilized in all cases, together with either a 36 mm or 42 mm glenosphere made of cobalt–chromium alloy. An appropriately sized humeral liner of standard polyethylene was utilized.

Post operation, all patients wore a sling for 5 to 6 weeks. A structured physiotherapy programme was commenced on the first postoperative day.

### 2.5. Statistical Methods

The data were analysed using a mixture of parametric procedures (*t*-tests and general linear models, including analysis of covariance) and nonparametric procedures (Mann–Whitney test, sign test, Fisher’s exact test and logistic regression), as appropriate. Analyses were conducted using either Minitab statistical software version 18 (Minitab, Inc., State College, PA, USA) or R (R Core Team) [23].

## 3. Results

**Figure 1:**
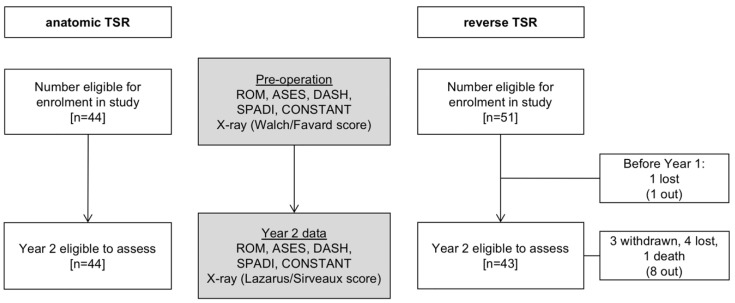
The CONSORT flowchart of the study. Pre- and post-operative assessment of range of motion (ROM), ASES, DASH, SPADI and Constant scores, and radiologic assessments.

The more than 2-year results of 44 patients with aTSR and 51 patients with rTSR were analysed (Figure 1). The two groups were fairly similar demographically, in particular they had similar preoperative VAS, ASES, SPADI, DASH, and CS scores. The average age of aTSR patients was 77.33 ± 1.97 and of rTSR 82.10 ± 3.93 years (Table 1). In the aTSR group, 72% were female compared with 90% in the rTSR group. In the aTSR group, glenoid morphology from the Walch classification was 16 A1, 11 A2, 12 B1 and 5 B2. There were no B3 or C or D glenoids. Glenoid assessment from the Favard classification for the rTSR showed 17 E0, 11 E1, 6 E2, 15 E3 and 2 E4.

All 44 included patients for aTSR completed the 2-year follow-up (Figure 1). In the rTSR group, eight patients were not available at 2 years, resulting in 43 patients at this time point.

Prospectively collected preoperative and 2-year postoperative clinical assessments, ROM, and postsurgery satisfaction, are presented in Table 2. The preoperative and 2-year postoperative VAS pain scores, CS, and PROMs are presented in Table 3.

At 2 years, while the final scores had improved for both groups, the aTSR group had better results. The final VAS pain scores for both groups were less than one on the pain scale, with the aTSR group having slightly less pain. The ROM had improved (*p* = < 0.001) with active elevation up to 147 degrees for aTSR and 125 degrees for rTSR. Patient satisfaction was significantly higher for aTSR than for the rTSR group, at 97.5% versus 90.09% (*p* < 0.001), respectively. The improvement from the preoperative scores was overall better in the aTSR than the rTSR group (Table 3). The final CS in the aTSR group had improved 46 points to 75 compared with the rTSR group’s improvement of 33 points to 56. The ASES showed comparative results (Table 3 and Table 4).

### 3.1. Radiologic Assessment

Radiolucency on the humeral side was only minor and did not differ between the groups. It was detected in three cases for the rTSR group and in four cases for the aTSR group at the 2-year mark (Table 5). The glenoid component demonstrated more surrounding radiolucency/notching signs for the rTSR (*n* = 10) than the aTSR group (*n* = 6) after 2 years. There was no significant component loosening in either group, and no dislocations, hence no revision surgery was required in either group.

### 3.2. Complications

There were no readmissions in either group. Complication rates were higher for the rTSR (*n* = 18 in 14 patients) than for the aTSR group (*n* = 3 in 3 patients) (Table 6).

One common complication seen in both groups was acromioclavicular joint (ACJ) pain. This occurred in two shoulders with an aTSR and in six shoulders with an rTSR. Most patients recovered well after a steroid injection, however in two cases (one case in each group), an arthroscopic excision of distal clavicle (EDC) was necessary with a good result.

One patient with an aTSR (Table 6) developed cuff failure. The MRI scan demonstrated a massively retracted and atrophied supraspinatus tendon by the time of presentation. As the shoulder joint was stable, AE was good (>90°) and there was no pain throughout the 2-year follow-up period, no revision surgery was undertaken.

Apart from the ACJ pain, the main other complication for the rTSR group was a stress fracture of the acromion or the scapular spine (*n* = 11), which mostly occurred within the first year, after patients began unrestricted use of the arm (Table 6). All cases resolved nonoperatively with rest for up to 3 months in an abduction pillow. One rTSR case had an avulsion fracture of the posterior inferior glenoid by the long head of the triceps 10 months after surgery. There was no functional deficit and only mild pain, which resolved after 2 months with nonoperative treatment.

## 4. Discussion

The current study compares patients following an aTSR versus an rTSR aged 75 years and older. The baseline demographics of the two groups were similar, including PROMs. The overall outcomes in the 2-year follow-up period confirm our original hypothesis, as better ROM, clinical outcome scores including patient satisfaction, and less complications were demonstrated for the aTSR group than the rTSR group, as shown in Table 4.

These results differ to Kiet et al. who looked at outcomes of aTSR and rTSR results after 2 years [24]. They demonstrated no differences in the complication or revision rates, nor ASES and pain scores. The ROM in both groups was similar except for a slightly better external rotation in the aTSR group, which was also found in our study. In addition, in our study, the AE was also significantly better in the aTSR group (*p* < 0.001). It is difficult to compare our results with Kiet’s study, as the average age of the patients is not documented, and further, they used a stemmed anatomic humeral prosthesis which was cemented and a metal humeral head [24].

Another study by Wright et al. compared aTSR with rTSR in patients aged 70 years and older with an intact rotator cuff [25]. They were able to identify similar postoperative ROM and outcome scores for both prostheses. Although they reported no significant differences in complication rates for the two prostheses types, they did identify that rotator cuff tears were the principal complication in the aTSR group leading to conversion to an rTSR, which occurred within an average of 28 months postsurgery. In the current study, there was one early cuff failure following an aTSR within 24 months follow-up, but revision of the prosthesis was not required.

An additional problem with an rTSR is that the medical complication rate has been reported to be significantly higher than with an aTSR [26]. The factor of age and complications was analysed by Koh et al. [27]. They assessed patients at 30 days postoperation and demonstrated that the older age group (>80 years) had significantly more complications and readmissions than younger patients for any type of shoulder arthroplasty [27].

Although all patients were 75 years of age and older, the rTSR patients were slightly older and a slightly lower ROM preoperatively, however, the PROMs and CS were very similar. Therefore, both groups started from a similar basepoint for all indices. However, the overall improvement in most indices and scores was greater in the aTSR group than the rTSR group. This resulted in the rTSR group having lower final postoperative clinical scores and a worse ROM than the aTSR group. It was not possible to analyse males’ versus females’ results due to the relatively few male patients.

Friedman and colleagues demonstrated that older patients with an rTSR had better outcome scores (ASES and SPADI) despite smaller improvement in abduction and forward flexion than younger patients [28]. Triplet et al. were able to demonstrate good improvements in motion (ER and AE), pain and function (ASES) in a smaller cohort of older patients (>80 years) for both types of shoulder replacements (aTSR *n* = 18 and rTSR *n* = 33). However, they did detect more postoperative complications and higher transfusion rates for rTSR patients [29]. This was different to Anakwenze and colleagues, who looked at the effect of age and outcomes in aTSR and rTSR [30]. They identified a higher odds ratio (OR) for patients older than 75 years for readmissions and an increased 1-year mortality in patients with an aTSR (OR 1.75 and 3.34) versus rTSR (OR 0.68 and 0.92). Furthermore, their hazard ratios (HR) for risk of revision were significantly lower for rTSR vs. aTSR patients in patients older than 75 years versus patients 75 years or younger, at HR 0.45 versus 1.24, respectively [30]. In our study there were no revisions. Another study from Wagner et al. demonstrated that no matter what sort of shoulder prosthesis is implanted, the risk of revision for mechanical failure, aseptic loosening or infection decreased with age 65 years and above [31]. According to their data, only instability remained an age-independent complication.

Stress fractures were the main complications seen in our rTSR group (21.6%), while none of the above-mentioned studies reported acromial or scapular stress fractures [25,26,32,33]. However, Zmistowski et al. reported an incidence of 10.5% for acromial stress fractures and reactions following rTSR, with an average follow-up of 407 days [34]. There was also no mention in the previous studies of acromioclavicular joint pain, as was seen in some cases in our study [25,26,32,33,34].

In our study, component loosening was not an issue in this short-term follow-up period. On radiographic evaluation of the humeral component side, there was no difference between prosthesis types, with only minor osteolysis seen for both the stemless aTSR and the long-stem rTSR. On the glenoid side, osteolysis for both types of component was again only minor, but there was a slight tendency towards more osteolysis with the rTSR than the aTSR, although it is difficult to compare in such different prostheses as notching is only seen with rTSR and radiolucent lines with aTSR.

Shields and Wiater [33] compared aTSRs which were revised to rTSRs because of rotator cuff failure or component loosening to matched patients with primarily an rTSR. They demonstrated that patients with a revision surgery for a failed aTSR not only had a lower satisfaction but also significantly poorer subjective outcome scores and more complications than the primary rTSR group. However, revision of a primary aTSR to an rTSR solely due to cuff failure has also been shown by Flury et al. to be a good salvaging procedure, as it improves ROM, clinical scores and patients’ satisfaction when comparing pre- and post-operative scores [32]. The latter study results support a surgeon’s decision to continue using an aTSR in older patients, especially as with a stemless prosthesis, revision of the components to an rTSR is relatively easy.

Despite prospective data collection, our study has limitations. The follow-up time is limited to 2 years, which is less than the average time of secondary rotator cuff failure in aTSR [25,35]. Despite this, during this short follow-up time, the rTSR demonstrated more complications, in particular stress fractures of the scapula, than aTSR. Patients receiving an rTSR were slightly older than the aTSR patients, skewing the data slightly, as there is a naturally increased chance with age of a later rotator cuff tear, which requires an rTSR rather than an aTSR [36]. Another limiting factor is the relatively small cohort size and the predominance of female patients, both of which are probably related to the increased age of the patients. Further research and longer follow-up studies need to be performed in order to address these issues.

## 5. Conclusions

Our results suggest that judicious patient selection in the older-aged patient (75+ years) for the type of shoulder replacement needs to be performed individually and not merely on the basis of patient age. This process can then lead to higher selection towards aTSR, which demonstrated in the current setting significantly better ROM and clinical outcomes than same-aged patients with an rTSR. Additionally, the complication rate is less for an aTSR in the first 2 years following shoulder replacement, making this an acceptable surgical option despite increased age. However, this study only has a short follow-up of 2 years, therefore the outcomes are to be judged carefully and preliminarily, as further prolonged research is necessary.

## Figures and Tables

**Table 1:** Patient demographics and preoperative radiographic glenoid scores (Walch [14,15] and Favard [16] classification).

Anatomic TSR		Reverse TSR	
	*n* = 44		*n* = 51
**Age** (years ± SD) (min; max)	77.37 ± 1.97 (75; 81)	**Age** (years ± SD) (min; max)	82.10 ± 3.93 (76; 91)
**BMI** (% ± SD) (min; max)	29.03 ± 4.82 (20.7; 40.8)	**BMI** (% ± SD) (min; max)	26.95 ± 4.01 (18; 37)
**Gender**		**Gender**	
male	12 (27.27%)	male	5 (9.80%)
female	32 (72.73%)	female	46 (90.20%)
**Operated arm**		**Operated arm**	
right	29	right	31
left	15	left	20
**Dominant arm**		**Dominant arm**	
right	40	right	47
left	4	left	4
**Walch classification**		**Favard classification**	
A1	16	E0	17
A2	11	E1	11
B1	12	E2	6
B2	5	E3	15
B3, C, D	0	E4	2
Missing	0	Missing	0

**Table 2:** Clinical range of motion assessment—external rotation (ER), active elevation (AE), stabilized scapular glenohumeral abduction (GH)—preoperative and at the 2-year follow-up mark and postsurgery satisfaction. The difference for each category from preoperative to 2-year postoperative is demonstrated with a delta (∆). (Mann–Whitney and two-sample *t*-test: * *p* < 0.05; ** *p* < 0.01; *** *p* < 0.001).

	Anatomic TSR			Reverse TSR		
	Mean ± SD (min; max)	*n*	Missing	Mean ± SD (min; max)	*n*	Missing
preOP ER [°]	17.79 ± 13.49 (−5; 50)	34	10	25.95 ± 22.09 (0; 80)	42	9
preOP GH [°]	52.32 ± 19.91 (10; 90)	41	3	55.54 ± 18.26 (10; 90)	46	5
preOP AE [°]	84.42 ± 31.80 (15; 150)	43	1	71.67 ± 32.90 (0; 130)	48	3
Y2 ER [°]	61.14 ± 14.10 (30; 90) ***	44	0	39.08 ± 15.50 (10; 70)	49	2
Y2 GH [°]	75.68 ± 12.32 (50; 90)	44	0	75.20 ± 10.05 (45; 90)	51	0
Y2 AE [°]	146.93 ± 18.84 (100;175) ***	44	0	125.29 ± 21.85 (90; 160)	51	0
Satisfaction (%)	97.5 ± 7.35 (60; 100) ***	44	0	90.09 ± 13.23 (50; 100)	45	6
∆ER (Y2—preOP)	42.50 ± 19.55 (0; 90) ***	34	10	12.50 ± 24.23 (−40; 50)	40	11
∆GH (Y2—preOP)	23.05 ± 23.37 (−30; 80)	42	3	19.89 ± 17.56 (−10; 50)	46	5
∆AE (Y2—preOP)	61.98 ± 33.19 (0; 135)	43	1	55.42 ± 30.94 (0; 140)	48	3

**Table 3:** Patient assessment with VAS pain levels, ASES, DASH, SPADI and Constant scores preoperative and 2 years postoperative. The difference for each category from preoperative to 2 years postoperative is demonstrated with a delta (∆). (Mann–Whitney and two-sample *t*-test: * *p* < 0.05; ** *p* < 0.01; *** *p* < 0.001).

	Anatomic TSR			Reverse TSR		
	Mean ± SD (min; max)	*n*	Missing	Mean ± SD (min; max)	*n*	Missing
preOP VAS pain (0–10)	5.62 ± 2.57 (1; 10)	42	2	5.73 ± 2.29 (2; 10)	51	0
preOP ASES Total	39.58 ± 20.03 (3; 73.33)	42	2	35.50 ± 17.38 (0; 63.33)	50	1
preOP SPADI Total	65.05 ± 20.96 (23.85; 97.69)	40	4	69.59 ± 15.94 (34.60; 100)	34	17
preOP DASH Total	49.80 ± 19.08 (15; 95) ***	40	4	64.16 ± 15.47 (30.83; 89.17)	38	13
preOP Constant Total	28.20 ± 12.93 (4; 60)	41	3	22.59 ± 13.40 (2; 58)	39	12
Y2 VAS pain (0–10)	0.29 ± 0.85 (0; 5)	44	0	0.56 ± 1.08 (0; 4)	45	6
Y2 ASES Total	90.58 ± 9.88 (63.33; 100) ***	44	0	73.50 ± 16.71 (30; 100)	39	12
Y2 SPADI Total	5.33 ± 8.25 (0; 36.92) ***	44	0	26.03 ± 19.83 (0; 84.62)	40	11
Y2 DASH Total	10.42 ± 10.65 (0; 48.33) ***	44	0	35.01 ± 22.35 (1.79; 80)	39	12
Y2 Constant Total	75.20 ± 11.41 (42; 96) ***	44	0	56.14 ± 11.48 (25; 76)	37	14
∆VAS pain (Y2—preOP)	−5.33 ± 2.68 (−10; −1)	42	2	−5.13 ± 2.38 (−10; −1)	45	6
∆ASES (Y2—preOP)	50.99 ± 20.67 (7; 90.33) **	42	2	39.17 ± 17.78 (0; 71.67)	38	13
∆SPADI (Y2—preOP)	−59.56 ± 21.28 (−95.85;−18.23) **	40	4	−44.34 ± 22.27 (−93.85; 4.62)	30	21
∆DASH (Y2—preOP)	−38.80 ± 18.86 (−89.33; −0.81)	40	4	−30.11 ± 22.16 (−79.17; 13.40)	33	18
∆Constant (Y2—preOP)	46.20 ± 17.44 (7; 77) **	41	3	33.39 ± 15.31 (4; 70)	33	18

**Table 4:** Overview and comparison of 2-year follow-up outcomes (radiological and clinical outcomes, patient satisfaction and complication rates). The “+” (plus sign) represents higher values in comparison with the other category, whereas “=” (equal sign) represtents no significant differences. (External rotation (ER), active elevation (AE), stabilized scapular glenohumeral abduction (GH)).

Categories	Anatomic TSR		Reverse TSR
Glenoid radiolucency/notching		=	
Humeral radiolucency		=	
ASES	+		
SPADI	+		
DASH	+		
Constant	+		
VAS pain		=	
ER	+		
GH		=	
AE	+		
Satisfaction	+		
Complication rate			+

**Table 5:** Postoperative radiologic assessment for the glenoid and humeral components for the anatomic and reverse TSR. Radiolucency for the glenoid component of aTSR is scored according to Lazarus [18], whereas for the rTSR, glenoid notching is scored according to Sirveaux [20].

Anatomic TSR			Reverse TSR		
**X-ray—Lazarus glenoid radiolucency score**	**Y1**	**Y2**	**X-ray—Sirveaux glenoid notching score**	**Y1**	**Y2**
Total eligible	44	44	Total eligible	50	43
0	39	38	0—No defect	43	33
1	5	6	1—Defect only concerns the pillar	6	8
2			2—Contact with the lower screw	1	2
3			3—Extension over the lower screw		
4			4—Extension under baseplate		
5					
Missing	0	0	Missing	0	0
**X-ray—humeral radiolucency score (zones)**	**Y1**	**Y2**	**X-ray—humeral radiolucency score (zones)**	**Y1**	**Y2**
Total eligible	44	44	Total eligible	51	43
No radiolucency	41	40	No radiolucency	49	40
Radiolucency cases (all zones)	3	4	Radiolucency cases (all zones)	2	3
Zone 1	1	2	Zone 1	0	1
Zone 2	0	0	Zone 2	0	0
Zone 3	0	0	Zone 3	0	0
Zone 4	0	0	Zone 4	0	0
Zone 5	2	2	Zone 5	0	0
			Zone 6	0	0
			Zone 7	2	3
Missing	0	0	Missing	0	0
**Drop-outs**	0	0	**Drop-outs**	1	8

**Table 6:** Postoperative complications for both types of shoulder replacements. Acromioclavicular joint (ACJ) pain is common among aTSR and rTSR, and can be resolved by injections or arthroscopic excision of the distal clavicle (EDC). The main complications for rTSR are stress fractures (#) of the acromion or the scapular spine. The category “Other” reports one case with an avulsion fracture of the triceps. Percentages (%) are based on total number of patients available at 2-year follow-up (aTSR *n* = 44; rTSR *n* =43).

Anatomic TSR			Complications	Reverse TSR		
*n*	%	Time ± SD after Surgery (Months)		*n*	%	Time ± SD after Surgery (Months)
2	4.54	18 ± 8.5	ACJ pain	6	13.95	13.5 ± 9.7
1	2.27	12	resolved by injection	5	11.62	11.4 ± 9.2
1	2.27	24	resolved by EDC	1	2.32	24
0	-	-	acromial and scapular stress #	11	25.58	9.9 ± 7.0
0	-	-	Instability	0	-	-
1	2.27	10	Cuff failure	0	-	-
0	-	-	Infection	0	-	-
0	-	-	Other	1	2.32	10
**3**	**6.81**	**10.0 ± 9.3**	**Total patients**	**14**	**32.55**	**11.7 ± 8.1**

## Data Availability

Data available on reasonable request due to privacy and ethics board restrictions. The data presented in this study are available on reasonable request from the corresponding author. The data are not publicly available due to privacy and ethics board restrictions.
